# Shifting to Delivering Simulation Virtually Within a Healthcare Education Setting

**DOI:** 10.7759/cureus.21598

**Published:** 2022-01-25

**Authors:** Eva Peisachovich, Nureen Ladha, Zipora Rahmanov, Celina Da Silva

**Affiliations:** 1 Medical Education and Simulation, York University, Toronto, CAN; 2 Nursing, York University, Toronto, CAN

**Keywords:** teaching technology, simulation in medical education, virtual learning, healthcare education, teaching by simulation

## Abstract

The coronavirus disease 2019 (COVID-19) pandemic has changed how healthcare education is being delivered, creating a global shift towards virtual modalities. Various approaches, each with their own benefits and limitations, have been developed to bridge this gap and continue to provide comprehensive education to healthcare students. To understand which approach to implement, we must consider what each can offer and what is best suited for the situation. Much of this will focus on the learning goals and outcomes with research strongly favouring modalities focused on the areas of cognitive, affective, and behavioural skill acquisition as opposed to technical skills.

The use of simulated persons offers the most diverse application for these areas of focus. This approach can provide opportunities for both synchronous and asynchronous learning. While novel in its approach, virtual simulations can leverage existing success and performance indicators used for in-person approaches to best understand the experiences of the learners and the facilitators. Evidence can be compared with outcomes of previous in-person groups to understand how this approach can be best implemented into curricula/programs.

Future applications are numerous for this modality and the development of pilot studies focused on smaller groups of learners will provide opportunities for educators and program developers to review and understand the challenges that may arise.

Simulation is a widely drawn upon teaching-learning approach deeply rooted in experiential learning. With the purpose of replicating real-world scenarios to increase knowledge transfer and reduce the shock of encountering challenging real-world situations, simulated environments are proven to be an effective tool in building learners' self-confidence and bridging the gap between theory and practice within the healthcare realm. Simulation can be, and is, applied within healthcare-education contexts from undergraduate to graduate levels and into ongoing professional development.

## Editorial

Transitioning to virtual simulations within healthcare education

The coronavirus disease 2019 (COVID-19) pandemic has created a necessary shift in healthcare-education strategies from in-person to virtual modalities [1]. Various virtual-simulation approaches have been adopted into healthcare education, each with beneficial applications to undergraduate, graduate, and ongoing professional levels of education in nursing, medicine, and midwifery. Each of these disciplines may use any combination of approaches; however, some modalities may have benefits over others depending on various contextual factors.

Virtual simulation games (VSGs) are one such approach; video-based clinical scenarios are the foundation for these interactive games. VSGs allow learners to engage with the material synchronously or asynchronously and have been found to have similar outcomes of living simulations.

Within a healthcare-education context such as medical and nursing curricula, telesimulations provide students with an opportunity to continue to partake in learning using high-fidelity scenarios and simulation equipment. This approach can be implemented in a few ways. In one, facilitators may use technology such as a mannequin that students would be able to view through a camera and access remotely to observe the technical skills being performed by the facilitator, such as vital sign monitoring, interventions, and physical assessment [2]. This approach focuses on the cognitive and behavioural skills by critically considering why actions are performed and the education provided to patients and families about the interventions, as opposed to the hands-on technical skills of performing the action [2]. Another approach using telesimulation focuses on assessing skills and clinical competencies [1]. Telesimulated Objective Structured Clinical Examinations (OSCEs) are conducted to evaluate student learning, clinical judgement, and professional conduct; during OSCEs, students demonstrate competencies in obtaining and assessing clinically relevant information, providing patient education, communicating important information, and developing a care plan [1]. While each educational institution may implement telesimulations differently, many have chosen to approach this experiential learning with preparatory information; for example, learners may be provided with the history and background of the simulated patient before entering the simulation [1,2]. Using online platforms such as Zoom, learners enter the virtual simulation where they are able to engage with the simulated patient [1,3]. Similar to in-person modalities, the simulation ends with a debrief that promotes self-reflection and critical thinking that can be applied to ongoing learning [1]. Variations of this approach include permitting the learner or facilitator to “time-out” during the simulation for feedback, in order to debrief and/or dissect important information and dialogue being captured during the session [3].

Simulated persons (SPs), also referred to as simulated patients within a healthcare context, can also be used in healthcare education. This application has many rich and innovative avenues to explore within a virtual context. In addition to the OSCEs as outlined above, SPs may be employed in telehealth or on-call simulations, wherein learners simulate virtual encounters similar to those they may encounter in real-life settings. This methodology uses persons trained in simulation to portray a role and create high-fidelity and immersive situations in which the learner is able to explore dynamic critical thinking and problem-solving skills [3]. Within a virtual context, SP methodology can be used either synchronously or asynchronously. Asynchronous applications provide learners with preparatory information and access to prerecorded simulations created using trained SPs, and also afford opportunities for online and synchronous debriefing [4]. Synchronous applications of SPs include the use of virtually simulated telephone triage. These synchronous and asynchronous simulations support the acquisition of telehealth and telephone-triage skills, which allow patients to receive healthcare while they remain in their homes [1]. These are essential skills to master, especially when considering those who cannot come into office settings to receive care due to mobility, financial constraints, or other social determinants of health.

Recommendations and considerations for the application of virtual simulated persons

As can be seen, there are various approaches to implementing virtual simulation within the healthcare-education context. When considering high-fidelity options, it is clear that using trained SPs offers the most diverse applications to various areas within the healthcare setting. Within a virtual-simulation context, research shows a stronger preference for applications focused on cognitive or affective learning outcomes than for those that are skills-based.

When considering the application of virtual SPs, the learning objectives are important to understand. Where self-directed study approaches are leveraged, such as continued professional development or graduate/post-graduate level studies, asynchronous virtual simulations, including VSGs, may be the best fit. However, while a virtual simulation is more cost-effective and can be supported for more significant learners [3], consideration should be given to the types of feedback and debriefing models available with this asynchronous approach.

Simulations in any setting must be meaningful to the learner to ensure the best learning outcomes [3]. Involving stakeholders (including learners) in the development of scenarios allows learners to meaningfully engage with scenarios more realistic to their experiences [4]. Providing learners an opportunity to become involved in developing scenarios that are similar to situations they may encounter in their practice settings may further engage learners and empower them, thus furthering concepts of learner-driven pedagogies and philosophical underpinnings.

While many of these approaches may have been born out of a necessity due to the pandemic, it is important to consider ongoing opportunities to leverage these technological approaches. Virtual simulations, both synchronous and asynchronous, show promise in limiting costs associated with travel, space rental, and human resources, while also allowing these approaches to reach learners beyond typical geographical boundaries [1,3]. Consider previous student placements for various healthcare students, which were bound by geographical constraints. Now, with virtual simulated persons, telesimulations, and on-call simulations, learners have the opportunity to engage with teachers and facilitators all over the world. Moreover, learners can now be exposed to areas that would otherwise be deemed too high-risk to allow students to engage in a meaningful way. Take, for example, placements in pediatrics or critical care; while placement opportunities are limited or high-risk for student learners, virtual simulations can accommodate students in a safe environment that allows them to engage with complex and otherwise unforeseen challenges [1].

Success indicators

The goal of simulation education is to enhance learning [3]. As virtual SPs are a novel approach to healthcare education, success indicators continue to emerge. Some of these indicators may include financial or other fiscal benefits, while others may be similar to those associated with in-person simulation approaches. Opportunities for learners to safely practice rarely encountered or difficult skills, develop self-confidence, and learn from mistakes before being expected to apply these skills to emergencies that may occur at the bedside are examples of this. Success indicators within an OSCE context would be specific to the learning objectives of the scenario. The success of virtual simulations has been assessed by applying validated and reliable tools such as the Simulation Effectiveness Tool; the findings include consolidation of learning and increases in confidence, clinical knowledge, critical thinking, and clinical judgement [5]. 

Limitations and recommendations

Successful simulation within education is predicated on its realism and on the learners’ willingness to buy into the approach. Within a virtual context, this becomes increasingly challenging. It is up to the educational institution or healthcare organization to commit to a level of realism that engages learners and allows them to become immersed in the simulation. The realism of the learning environment must match, as best as possible, to the real-world settings learners are likely to encounter.

Additionally, while the in-person SPs approach continues to be cost-effective, the same cannot be said for high-fidelity simulation approaches that require specific equipment to demonstrate skill acquisition. In many cases, the institution pays for this teaching modality using tuition fees or grants while in some cases, students pay for the virtual simulation. While students paying tuition and attending in-person skills labs would have had access to tools provided by the institution, it remains to be seen whether students participating in virtual simulations, despite paying the same tuition, would be expected to take on this additional cost. There may also be a need to consider policy implementation to support the use of virtual simulated persons within healthcare education beyond the pandemic. How may policy provide the foundation for additional funding to support this well-proven modality? When considering scenario development, should there be regulations surrounding ethical implications of scenarios that may be triggering? Are there ethical implications to consider for high-fidelity and immersive scenarios?

Perhaps the most pressing question is, are these virtual simulations sufficient to demonstrate learning? For example, in the case of OSCEs, learners are not able to conduct physical exams. To overcome some of these barriers, virtual simulated-person scenarios should focus on the cognitive aspects of skill acquisition and on the why as opposed to the actions themselves [2]. Examples could include recognizing warning signs of a worsening condition rather than engaging in the physical assessment that would be conducted in that situation. This becomes especially important when considering telephone-triage skills, where physically assessing a patient is not feasible, and much of the assessment is based on asking appropriate questions to recognize a possible urgent or emergent situation.

Relying on technology comes with inherent challenges. For example, limitations to the field of vision may only allow parts of the body to be visible in the frame, which may limit access to other non-verbal cues [3]. Additionally, depending on the location of the camera of both learner and facilitator, eye contact may not be aligned with camera angles [3]. Finally, a reliable internet connection is necessary; otherwise, interruptions in inflow and learning could negatively impact the learner’s ability to buy into the scenario.

Future directions 

As highlighted, virtual simulation using SPs has many prospective applications to healthcare education and continuing education. Offering both synchronous and asynchronous opportunities to participate in the simulation, combined with synchronous debriefing sessions, provides autonomy to learners. Focusing both on high-fidelity scenarios in which facilitators modify environments to most closely emulate real-world settings and on cognitive, behavioural, and affective skill demonstration that uses scenarios developed in collaboration with the learners may create the most meaningful experience for learners. Scenarios should include opportunities to explore areas of nursing in which placements are limited or high-risk to increase learners’ exposure to these areas. As we move forward in a technology-driven and hybrid healthcare model, virtual simulations using SPs should focus on the future virtual application of healthcare, including supporting homebound patients, telehealth, and on-call triages to recognize and assess urgent and emergent health concerns. Having feedback models in place to receive input from students and faculty will be important in building robust and integrated programs/curricula. Developing pilot studies focused on smaller groups of learners may also provide an opportunity to understand the challenges that may arise with the methodology.

There are promising applications for virtual simulations on the horizon. As we move forward, we will need to continue innovating, exploring, and understanding how simulation education has shifted and how we, as educators and leaders, can implement this methodology to support students in their ongoing learning.

## References

[1] Bradford HM, Farley CL, Escobar M, Heitzler ET, Tringali T, Walker KC (2021). Rapid curricular innovations during covid-19 clinical suspension: maintaining student engagement with simulation experiences. J Midwifery Womens Health.

[2] Diaz MC, Walsh BM (2021). Telesimulation-based education during COVID-19. Clin Teach.

[3] Peisachovich E, Da Silva C, Penhearow NJ, Sombilon EV, Koh M (2020). Implementing virtual simulated person methodology to support the shift to online learning: technical report. Cureus.

[4] Da Silva C, Peisachovich E, Gal R, Anyinam C, Coffey S, Graham Graham, L L (2020). A programmatic approach to the design of a video simulation case study. Clin Simul Nurs.

[5] Kuszajewski ML, Vaughn J, Bowers MT, Smallheer B, Hueckel RM, Molloy MA (2021). Embracing disruption: measuring effectiveness of virtual simulations in advanced practice nurse curriculum. Clin Simul Nurs.

